# Loop-mediated isothermal amplification-microfluidic chip for the detection of *Trichophyton* infection

**DOI:** 10.3389/fmicb.2022.1031388

**Published:** 2022-10-13

**Authors:** Weiwei Jiang, Dongying Hu, Yanyan Xu, Yang Chen, Xiaoyang Zhu, Zhao Han, Xin Ye, Xiaojing Li

**Affiliations:** ^1^Department of Dermatology, 72nd Group Army Hospital of PLA, Huzhou, Zhejiang, China; ^2^Affiliated Hospital of Hebei University of Engineering, Handan, Hebei, China; ^3^Department of Laboratory Medicine, The First Affiliated Hospital of Xi’an Jiaotong University, Xi’an, Shaanxi, China

**Keywords:** *Trichophyton*, LAMP, microfluidics, early diagnosis, chip

## Abstract

*Trichophyton* is the most pathogenic type of fungal skin infection. It often invades and grows in a keratin-rich matrix, and lesions include human skin, hair, and fingernails (toenails). We designed LAMP primers for *Trichophyton* and developed a LAMP-Microfluidic chip detection system for *Trichophyton*. This system detects six common species of *Trichophyton* in the genus *Trichophyton*, including *Trichophyton rubrum*, *Trichophyton mentagrophyte*, *Trichophyton violaceum*, *Trichophyton tonsurans*, *Trichophyton verrucosum*, and *Trichophyton schoenleinii*. The specificity reached 100%, and the sensitivity could reach about 1 × 10^2^ copies/μl. The entire detection process can be completed within 60 min and does not cross-react with other dermatophytes. The established LAMP-Microfluidic chip detection system has the advantages of simple operation, high specificity, and high sensitivity, and has the potential for clinical application.

## Introduction

*Trichophyton* is the most common group of fungi responsible for superficial fungal infections, which affect 20–25 percent of the world’s population (Havlickova et al., 2008; Maraki and Mavromanolaki, 2016; Zhan and Liu, 2017). *Trichophyton* infects a large number of people and can cause tinea capitis, onychomycosis, tinea manuum, tinea pedis, tinea corporis, etc. It spreads easily, causing self-infection and infecting others. Coupled with irregular treatment and other reasons, it is easy to have a repeat infection. The lack of a long-term cure seriously affects the patient’s quality of life. In addition, tinea pedis and onychomycosis may be risk factors for developing acute leg cellulitis or erysipelas, especially in patients with diabetes (Bristow and Spruce, 2009). According to an epidemiological survey of large-scale superficial mycosis in China, the most common pathogenic species of *Trichophyton* are *Trichophyton rubrum*, *Trichophyton mentagrophyte*, *Trichophyton violaceum*, *Trichophyton tonsurans*, *Trichophyton verrucosum*, and *Trichophyton schoenleinii* (Shao-xi et al., 2011). It is recommended to carry out drug treatment from the level of *Trichophyton* (Fuller et al., 2001, 2014; Devliotou-Panagiotidou and Koussidou-Eremondi, 2004; Kakourou et al., 2010). Irregular antifungal treatment increases the difficulty of treating skin diseases, leads to recurrent attacks, prolongs the course of the treatment, and increases the economic burden on the patients. However, due to the lack of timely and accurate diagnostic measures, the current clinical treatment of dermatophytosis is generally only empirical treatment, such as tinea capitis or onychomycosis, which usually requires several months of antifungal treatment. Therefore, an accurate diagnosis is essential before initiating treatment (Feuilhade de Chauvin, 2005).

Traditionally, fungal microscopy and culture methods are used to detect *Trichophyton* infection, and direct microscopy results are highly subjective, with false-negative cases accounting for 15–30% of routine tests (Summerbell et al., 2005; Panasiti et al., 2006). When conidia or hyphae of strict saprophytic fungi are too shallow or too far from the onychomycosis sampling, they can be seen upon direct inspection of the sample, resulting in false-positive results (Gianni et al., 1997). Most *Trichophytons* species are cultured for a longer time (2 weeks). Some supplementary tests, such as urease, nutrients, and other biochemical tests, or *in vitro* hair perforation tests, are also carried out when identifying some *Trichophyton* species (Shadomy and Philpot, 1980). Therefore, it is particularly necessary to develop a rapid diagnostic method for *Trichophyton* with a high detection rate, high accuracy, low reagent cost, low instrument cost, simple operation, certain high throughput, and direct clinical significance.

Loop-mediated isothermal amplification (LAMP) technology is an isothermal nucleic acid amplification technology (Notomi et al., 2000). It has the advantages of high sensitivity, short reaction time, and simple operation. It has been widely used to detect various pathogens (Kasahara et al., 2014; Liu et al., 2016; Tang et al., 2016; Li et al., 2017). Centrifugal microfluidics labs (lab-on-a-chip) can concentrate various unit technologies on a chip, and finally realize the miniaturization and automation of the entire detection integration; due to the integrated function of the microfluidic chip, the pollution of the sample to the environment during manual operation is minimized; the microfluidic chip can design the number of sample tanks and reaction chambers according to needs, which greatly shortens the detection time and improves the detection efficiency compared with the traditional project-by-project detection; in addition, the system needs less detection reagents and test sample size (Madou et al., 2006; Gorkin et al., 2010).

In this study, the real-time LAMP technology was combined with the microfluidic laboratory-on-a-chip technology to achieve a high-throughput and rapid diagnosis of *Trichophyton*. The formulation of the correct clinical drug regimen is of great significance.

## Experimental

### Experimental strains and nucleic acid extraction

There were 111 strains in total, including 20 strains of *Trichophyton rubrum*, 20 strains of *Trichophyton mentagrophytes*, 11 strains of *Trichophyton violaceum*, 4 strains of *Trichophyton tonsurans*, 3 strains of *Trichophyton verrucosum*, 8 strains of *Trichophyton schoenleinii*, 16 strains of *Microsporum canis*, 3 strains of *Microsporum ferrugineum*, 6 strains of *Epidermophyton floccosum*, 5 strains of common *Candida*, 5 strains of *Malassezia*, 4 strains of *Aspergillus*, 2 strains of *Penicillium*, 2 strains of *Rhodotorula*, and 2 strains of bacteria (Table 1). The strain nucleic acid extraction method was a rapid nucleic acid extraction method. The one-step nucleic acid extraction technology was independently developed by the Shanghai igenetec diagnostics Co. Ltd. There was no need to open the lid during extraction, avoiding aerosol contamination. The nucleic acid extraction kit had a high nucleic acid extraction efficiency and fully met the requirements for DNA sequencing, the PCR reaction, and the isothermal amplification experiments. The entire extraction process could be completed within 15 min.

**TABLE 1:** List of strains used in the test.

Genus	Strain	Strain number
*Trichophyton*	*T. rubrum*	SCZ60001; SCZ60002; SCZ60006; SCZ60009; SCZ60093; SCZ60096; SCZ60097; SCZ60098; SCZ60099; SCZ600100; SCZ94501; SCZ94502 SCZ94503; SCZ94504; SCZ94505; SCZ94506; SCZ94507; SCZ94508; SCZ94509 ATCC-MYA-4438
	*T. mentagrophyte*	SCZ30008; SCZ30023; SCZ60092; SCZ60245; SCZ60339; SCZ94510; SCZ94511; SCZ94512; SCZ94513 SCZ94514; SCZ94515; SCZ94516; SCZ94517; SCZ94518; SCZ94519; SCZ94520; SCZ94521; SCZ94522; SCZ94523; SCZ94524
	*T. tonsurans*	SCZ30024; SCZ30025; SCZ60342; SCZ60344
	*T. violaceum*	SCZ30005; SCZ30039; SCZ30041; SCZ30046; SCZ30050; SCZ30052 SCZ30057; SCZ30061; SCZ30066; SCZ30068; SCZ60352
	*T. verrucosum*	SCZ30016; SCZ30019; SCZ30020
	*T. schoenleinii*	SCZ30007; SCZ60033; SCZ60035; SCZ60340; SCZ60349; SCZ60350; SCZ60351; SCZ60353
*Microsporum*	*M. canis*	SCZ60167; SCZ60168; SCZ60169; SCZ60170; SCZ60171; SCZ60172; SCZ60173; SCZ60174; SCZ60175; SCZ60179; SCZ94525; SCZ94526; SCZ94527; SCZ94528; SCZ94529; SCZ94530
	*M. ferrugineum*	SCZ30012; SCZ30014; SCZ30015
*Epidermophyton*	*E. floccosum*	SCZ30031; SCZ30032; SCZ60140; SCZ94531; SCZ94532; SCZ94533
*Candida*	*C. albicans*	ATCC MYA-2876
	*C. parapsilosis*	ATCC 22019
	*C. tropicalis*	ATCC 66029
	*C. glabrata*	ATCC 28226
	*C. krusei*	ATCC 2159
*Malassezia*	*Malassezia*	SCZ94534; SCZ94535; SCZ94536; SCZ94537; SCZ94538
*Aspergillus*	*A. fumigatus*	ATCC-MYA-3627
	*A. niger*	SCZ 10135
	*A. flavus*	SCZ 10138
	*A. terrestris*	SCZ60285
*Penicillium*	*Penicillium*	SCZ60287; SCZ60288
*Rhodotorula*	*Rhodotorula*	SCZ20005; SCZ20007
*bacteria*	*Staphylococcus epidermidis*	BP-1; BP-2

T, Trichophyton; M, Microsporum; E, Epidermophyton; C, Candida; A, Aspergillus.

### Primer design and screening

First, the genome sequences of common species of *Trichophyton* were retrieved from Genbank, and the target genes were found to be highly similar to those of the species by Geneious software for analysis and comparison. The selected target gene fragments were significantly different from the sequences of other genera or adjacent species on the phylogenetic tree. Then, using the LAMP primer design software primer ExplorerV4, the *Trichophyton* primers were designed (outer primers F3 and B3; inner primers FIP and BIP, and loop primers LB and LF can be added to further shorten the reaction time). A total of three sets of primers were designed, and their sequences are shown in Table 2.

**TABLE 2:** *Trichophyton* primer sequence list.

Primer number	Primer name	Primer sequence
1	F3	CCGTCGCTACTACCGATTG
	B3	TCTGCTCACCCTGATGGA
	FIP	CAACTTTCCGGCCCTGGGC-GTGAGGCC TTCGGACTGG
	BIP	TCCGTAGGTGAACCTGCGGA-ACG TCGGTCCCTATCGTG
2	F3	ACCGATTGAATGGCTCAGTG
	B3	TCTGCTCACCCTGATGGA
	FIP	TGACCAACTTTCCGGC CCTGAGGCCTTCGGACTGGC
	BIP	TCCGTAGGTGAACCTGC GGAACGTCG GTCCCTATCGTG
3	F3	ACCGATTGAATGGCTCAGTG
	B3	ACGTCTGCTCACCCTGATG
	FIP	TGACCAACTTTCCGGCC CTGAGGCCTTCGGACTGGC
	BIP	AGGTTTCCGTAGGTGAACCT GCGAACGTCGGTCCCTATCGTG

The components were thoroughly mixed according to the LAMP reaction system in Table 3 and amplified using a 9600Plus fluorescence PCR instrument. In the primer screening stage, the reaction temperature was set to 63°C for 60 s, 60 s is a cycle, and the number of cycles was 90. The amplification curve of the reaction was observed in real-time, and the selected primer and the corresponding cut-off value were comprehensively judged in combination with the CT value.

**TABLE 3:** Loop-mediated isothermal amplification (LAMP) reaction system.

Reactive components	Volume (μ l)
LAMP reaction buffer	17.7
FIP	0.40
BIP	0.40
F3	0.05
B3	0.05
LB (H_2_O)	0.20
LF (H_2_O)	0.20
Bst polymerase	1.0
DNA template	5
Total	25

### Loop-mediated isothermal amplification system evaluation and verification

#### Specific experimental testing and validation of the system

Using the 9600Plus fluorescence PCR instrument, according to the reaction system in Table 3, the experimental strain DNA was prepared according to the primer system after screening to prepare a reaction solution. The amplification test was carried out under the conditions of 63°C for 60 s, and the number of cycles was 60. The DNA concentration was adjusted to 2.5–5 ng/μl. The amplification curve, CT value, etc., were used to determine whether the reaction was amplified, the typical amplification curve of the system reaction is s-type, and the reaction time is determined by combining the CT value at this time. And then, the number of strains was expanded for the experiment and verification.

#### Sensitivity test and verification of the system

The *Trichophyton* strain DNA was diluted according to the concentration gradient of 10^5^ copies/μl, 10^4^ copies/μl, 10^3^ copies/μl, 10^2^ copies/μl, 10^1^ copies/μl, and negative control (H_2_O). The reaction conditions were the same as the specificity experiment, and the amplification curve and CT value were observed to determine whether the amplification reaction occurred, the typical amplification curve of the system reaction is s-type, and the reaction time is determined by combining the CT value at this time. The experiment was repeated to further verify the sensitivity of the system and obtain the lowest positive concentration before the cut-off value for *Trichophyton*.

### Evaluation and verification of the microfluidic chip system

#### Fabrication of the microfluidic chip system

**FIGURE 1:**
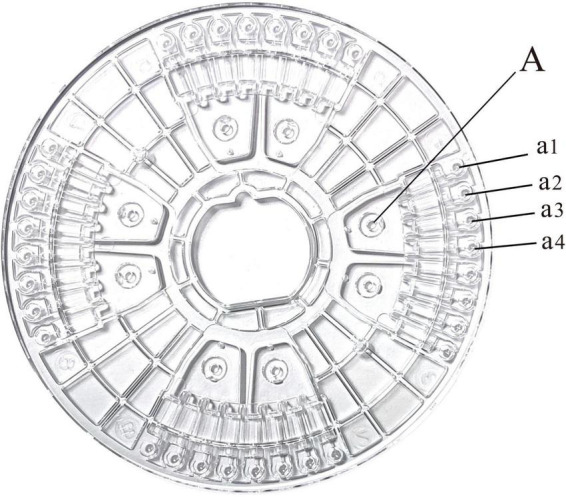
Microfluidic chip: (A) Sample tank; (a1–a4) reaction chamber.

In this experiment, a microfluidic chip was used, and the microfluidic chip was bought from Shanghai igenetec diagnostics Co. Ltd. (product number: CP01620NK). The microfluidic chip had 8 sample feeding slots, each of which corresponded to 4 reaction wells, for a total of 32 reaction wells (Figure 1). First, the microfluidic chip reaction plate was made. Then, the reaction solution for the microfluidic reaction well was prepared (Table 4 for the specific system). The sample volume of each reaction well was 1.5 μl, and each microfluidic chip had 32 sample wells. A total of 1.5 μl/well of the reaction solution was added to the reaction chamber, and the membrane was sealed for later use.

**TABLE 4:** Microfluidic reactor mix.

Reactive components	Volume (μ l)
FIP	0.08
BIP	0.08
F3	0.01
B3	0.01
H_2_O	1.06
0.1% trehalose	0.26
Total	1.50

The reaction system and reaction conditions of the sample addition tank are shown in Table 5. The sample volume of each sample addition tank was 50 μl. The amplification experiments were carried out using a microfluidic chip isothermal amplification nucleic acid analyzer. The reaction conditions were as follows: the temperature of the reaction hole was 63.0°C; the number of detection cycles was 60 (each cycle was 60 s); the low-speed centrifugal speed was 1,600 rpm; the low-speed centrifugal time was 10 s; the high-speed centrifugal speed was 4,600 rpm; and the high-speed centrifugal time was 30 s.

**TABLE 5:** Sample tank reaction system mix.

Reactive components	Volume (μ l)
Buffer	35.4
Bst polymerase	2
H_2_O	2.6
DNA template	10
total	50

#### Specific experiments for the microfluidic chip system

According to the reaction system in Table 5, the strain DNA was used to prepare the reaction solution. The sample DNA and negative control nucleic acid were added to the eight sample addition tanks of the microfluidic control according to the experimental plan, and the DNA concentration was adjusted to 2.5–5 ng/μl. After adding the sample, the sample was sealed with a sealing film and added to the microfluidic amplifier. The experiments were conducted according to the above reaction conditions. The amplification curve and CT value were observed to determine whether an amplification reaction occurred, the typical amplification curve of the system reaction is s-type.

#### Sensitivity experiment for the microfluidic chip system

The dermatophyte strain DNA was added into the reaction tank according to the diluted concentration gradient, and the gradient was 10^5^ copies/μl, 10^4^ copies/μl, 10^3^ copies/μl, 10^2^ copies/μl, 10^1^ copies/μl, and negative control (H_2_O). The reaction conditions were the same as the specificity experiment, and the amplification curve and CT value were observed to determine whether the amplification reaction occurred, the typical amplification curve of the system reaction is s-type. The experiment was repeated to further verify the sensitivity of the system and obtain the minimum amplification concentration before the cut-off value of *Trichophyton*.

## Results and discussion

### Loop-mediated isothermal amplification system specificity experimental results

The three groups of *Trichophyton* primers were screened for specificity. The amplification curves of the positive sample of primer No. 3 and the control sample were well distinguished, and the peak time of the control sample was the latest. Therefore, primer No. 3 was selected as the primer for *Trichophyton*. After the preliminary screening of several specific experiments, the positive samples were well amplified before 40 cycles, the negative samples in the optimized system were amplified after 60 cycles, and the positive amplification CT value was set to 40.0.

#### Loop-mediated isothermal amplification system specificity experimental results

**FIGURE 2:**
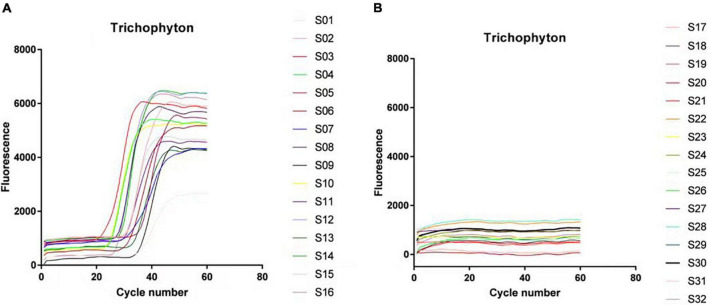
Loop-mediated isothermal amplification (LAMP) system specificity experimental results. **(A)** *Trichophyton* test results. **(B)** Experimental results of control strains.

There were 66 strains of *Trichophyton* in total, including 20 strains of *Trichophyton rubrum*, 20 strains of *Trichophyton mentagrophytes*, 11 strains of *Trichophyton violaceum*, 4 strains of *Trichophyton tonsurans*, 3 strains of *Trichophyton verrucosum*, and 8 strains of *Trichophyton schoenleinii*. These strains were all positively amplified before 40 cycles, and the negative strains, including dermatophytes of other genera and other common clinical fungi, bacteria, etc., did not amplify. The LAMP technology of this primer system had a diagnostic specificity of 100% for *Trichophyton*. Figure 2 is the amplification curve diagram. The positive samples S1–16 were: SCZ 60001; SCZ 60006; SCZ 60009; ATCC-MYA-4438; SCZ30008; SCZ30023; SCZ60092; SCZ30024; SCZ30005; SCZ30039; SCZ30016; SCZ30019; SCZ30007; SCZ60033; SCZ60035; SCZ60340. The negative samples S17∼32 were: SCZ60167; SCZ60168; SCZ60169; SCZ60170; SCZ60171; SCZ60172; SCZ30012; SCZ30014; SCZ30031; SCZ30032; ATCC MYA-2876; SCZ94534; ATCC-MYA-3627; SCZ60287; SCZ20005; BP-1.

#### Loop-mediated isothermal amplification system sensitivity test results

**FIGURE 3:**
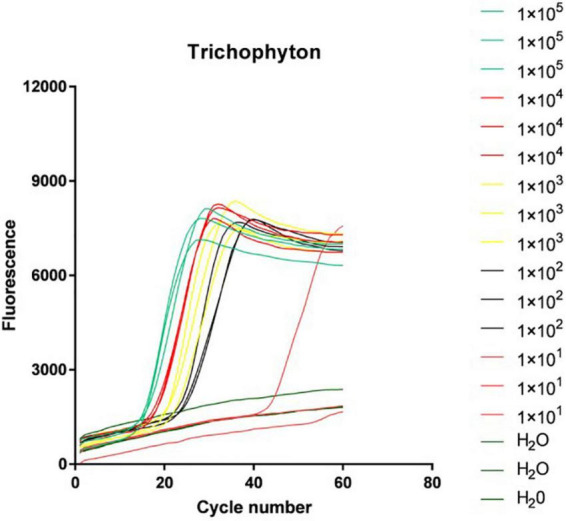
Loop-mediated isothermal amplification (LAMP) system sensitivity test results.

The *Trichophyton* DNA was sequentially diluted according to the concentration gradient and then added to the above LAMP reaction system. Figure 3 shows the isothermal amplification results of the ATCC-MYA-4438 strain samples, which were 1 × 10^5^ copies/μl 1 × 10^4^ copies/μl, 1 × 10^3^ copies/μl, 1 × 10^2^ copies/μl, 1 × 10^1^ copies/μl, and negative control (H_2_O). The samples were expanded, and the results were repeated for verification. The sensitivity of LAMP for verifying *Trichophyton* was 1 × 10^2^ copies/μl.

### Microfluidic chip system experimental results

The above-verified LAMP system was combined with a microfluidic chip to further verify the specificity and sensitivity of the system on the microfluidic chip platform.

#### Microfluidic chip system specificity experimental results

**FIGURE 4:**
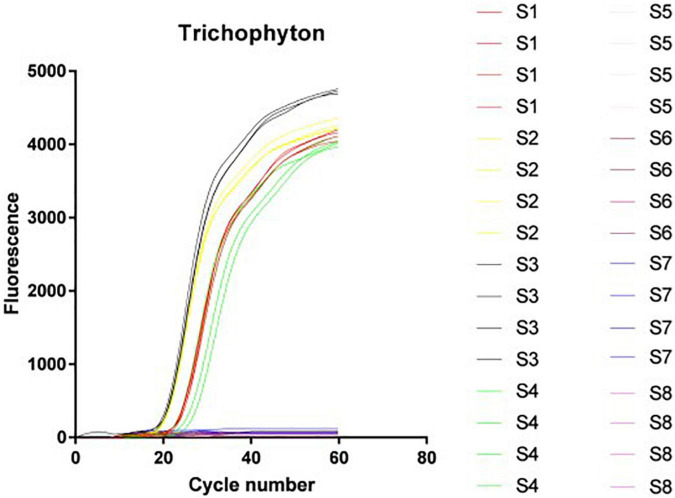
Microfluidic chip system specificity experimental results.

In this study, an 8-channel microfluidic chip was used as the reaction carrier, and each channel corresponds to four reaction wells (as shown in Figure 1). It was verified that the DNA of 66 *Trichophyton* strains showed positive amplification before 40 cycles, and the negative strains, including dermatophytes of other genera and other common clinical fungi, bacteria, etc., were not amplified. The amplification specificity of the *Trichophyton* primer system on the microfluidic chip was 100%. Figure 4 shows the amplification curve. The positive samples S1∼4 were: ATCC-MYA-4438; SCZ30023; SCZ30024; SCZ60340; The negative samples S5–8 were: SCZ60167; SCZ30014; SCZ30032; ATCC-MYA-3627.

#### Microfluidic chip system sensitivity experimental results

**FIGURE 5:**
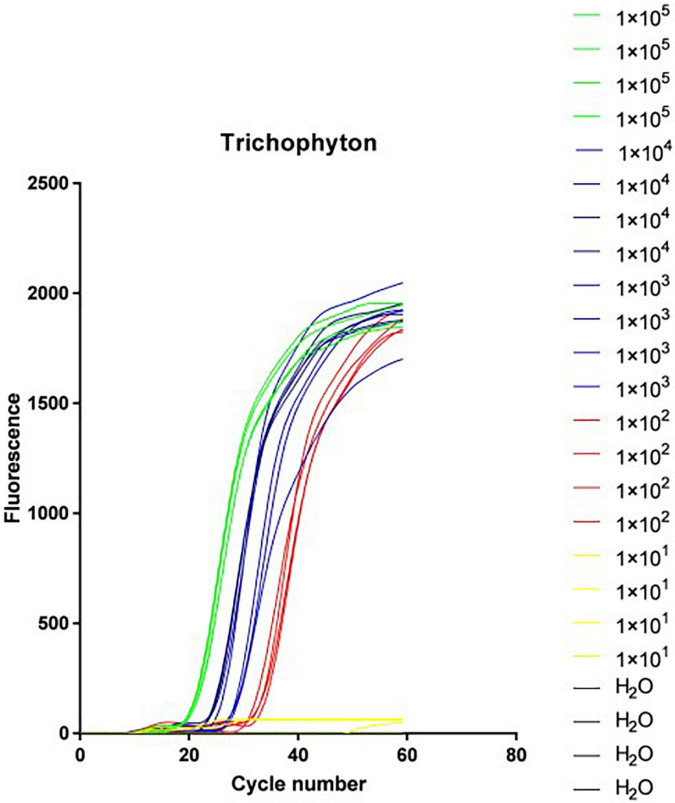
Microfluidic system sensitivity experimental results.

The *Trichophyton* DNA was sequentially diluted according to the concentration gradient and added to the reaction wells of the microfluidic chip. Figure 5 shows the isothermal amplification results of the strain ATCC-MYA-4438 samples, from left to right as follows: 1 × 10^5^ copies/μl, 1 × 10^4^ copies/μl, 1 × 10^3^ copies/μl, 1 × 10^2^ copies/μl, 1 × 10^1^ copies/μl, and the negative control (H_2_O). The amplified samples were the same as the repeated verification results. The sensitivity of LAMP for *Trichophyton* in the verification was 1 × 10^2^ copies/μl.

## Discussion

*Trichophyton* infection can affect the whole body, the skin, hair, finger (toe) nails, etc. Because of the difficulty in distinguishing *Trichophyton* infection from non-fungal dermatitis, especially dystrophic nails, in most cases, the laboratory helps confirm the diagnosis and initiate the appropriate treatment (Hainer, 2003). Recently, a series of molecular diagnostic methods for fungal infections, such as *Trichophyton*, have been gradually developed and applied in clinical practice. These mainly include two categories: one is based on skin plant protein diagnostic techniques, including Surface-enhanced Raman Spectroscopy (SERS), MALDI -TOF, etc.; the other is diagnostic technologies based on skin plant nucleic acids, including PCR product electrophoresis, multiplex PCR, PCR-TRFLP technology, PCR-ELISA detection method, real-time fluorescent quantitative PCR and isothermal amplification technology, etc. These early diagnostic methods allow for the identification of pathogenic microorganisms within hours with a high degree of specificity compared to traditional diagnostic methods, and the increasing use of MALDI-TOF mass spectrometry in the laboratory should facilitate the identification of microorganisms from cultured strains. The identification of dermatophytes, especially in the case of atypical isolates (L’Ollivier et al., 2013), by recent PCR-based studies has reported positive rates ranging from 74 to 100% (Verrier et al., 2013).

In a study including 60 patients, Rothmund et al. (2013) compared the efficacy of different methods for the diagnosis of onychomycosis. PCR had the highest sensitivity (90%). However, these techniques also have certain limitations. For example, a mass spectrometer (MALDI-TOF), which has been used clinically, is expensive with a high cost of maintenance, making it difficult to popularize. In addition, the current reference library of *Trichophyton* is insufficient, and changes in protein expression related to culture conditions may alter the MALDI-TOF MS results (L’Ollivier and Ranque, 2017). Multiplex PCR, because the same system contains multiple sets of primers, has a high possibility of primer-dimerization, leading to false positive results. Real-time fluorescent quantitative PCR has higher requirements for the experimental conditions and the operators. Therefore, despite the good sensitivity and specificity of many studies and faster PCR reactions, the routine diagnosis of these superficial fungal diseases currently relies on traditional methods (Petinataud et al., 2016). Therefore, there is an urgent need for an early diagnosis method that is both rapid and cost-effective.

This study combined the LAMP and microfluidic chip technologies, in which LAMP is a novel nucleic acid amplification method that can directly amplify specific DNA under isothermal conditions (Notomi et al., 2000; Nagamine et al., 2001). This technique eliminates the temperature cycling required for polymerase chain reaction (PCR), and most importantly, the method does not require denatured DNA templates (Nagamine et al., 2001) and can amplify 10^9^ copies of target DNA within 1 h (Notomi et al., 2000; Nagamine et al., 2001). The microfluidic chip technology was used to establish the above nucleic acid constant temperature amplification system on a miniaturized chip, design corresponding multi-channel reaction wells on the chip according to the experimental purpose and requirements, and pre-establish the reaction requirements on the channel. According to the recommendations of the latest edition of the “International Diagnosis and Treatment Guidelines,” using LAMP primer design software to design the corresponding genus primers from the level of *Trichophyton* species, requiring that the primers of the genus must be able to accurately identify common *Trichophyton* species, and can’t amplify dermatophytes of other genera and experiment-negative strains while meeting the characteristics of high amplification efficiency, good stability, and no cross-reaction. A total of 111 strains were included in this study, of which 66 were *Trichophyton* and 45 were control strains. After a comprehensive screening and alignment, a set of primers for the genus *Trichophyton* was selected. According to the results of multiple specificity experiments, the cut-off value of the system was determined to be 40 cycles, that is 40 min (one cycle in this study was 60 s). Then, the specificity and sensitivity of the system were tested. The results showed that the specificity of the LAMP technology for the identification of common dermatophytes of the genus *Trichophyton* reached 100%, and the sensitivity reached 1 × 10^2^ copies/μl. After verification, the reaction system was also well-verified on the microfluidic chip, with the same specificity and sensitivity as the LAMP technology. This diagnostic system greatly reduces the cost and time of testing a single patient, while increasing the number of patients tested per unit of time.

## Conclusion

In summary, this study successfully established a high-throughput, highly efficient, and low-cost detection platform for six species of *Trichophyton*, including *Trichophyton rubrum*, *Trichophyton mentagrophyte*, *Trichophyton violaceum*, *Trichophyton tonsurans*, *Trichophyton verrucosum*, and *Trichophyton schoenleinii*. The study revealed that the LAMP technology identified the common dermatophytes in *Trichophyton* with a specificity of 100% and a sensitivity of 1 × 10^2^ copies/μl. After verification, the reaction system was also well-verified on the microfluidic chip. The specificity and sensitivity were the same as the LAMP technology, and the whole process could be completed in 60 min. The LAMP-microfluidic chip is simple to operate, easy to popularize, and has a good application prospect for the detection and diagnosis of clinical *Trichophyton* infection.

## Data availability statement

The original contributions presented in this study are included in the article/supplementary material, further inquiries can be directed to the corresponding authors.

## Author contributions

WJ, DH, and YX conducted the experimental section, in which WJ also wrote the manuscript. YC and XZ were involved in strain identification and primer design. ZH and XY were responsible for experimental guidance and data analysis. XL was responsible for article design, experimental protocol adjustment, and manuscript review. All authors contributed to the article and approved the submitted version.
